# The Prolyl Isomerase Pin1 Modulates Development of CD8+ cDC in Mice

**DOI:** 10.1371/journal.pone.0029808

**Published:** 2012-01-04

**Authors:** Theresa J. Barberi, Alexis Dunkle, You-Wen He, Luigi Racioppi, Anthony R. Means

**Affiliations:** 1 Department of Pharmacology and Cancer Biology, Duke University Medical Center, Durham, North Carolina, United States of America; 2 Department of Immunology, Duke University Medical Center, Durham, North Carolina, United States of America; 3 Department of Cellular and Molecular Biology and Pathology, University of Naples Federico II, Naples, Italy; University Paris Sud, France

## Abstract

**Background:**

Pin1 has previously been described to regulate cells that participate in both innate and adaptive immunity. Thus far, however, no role for Pin1 has been described in modulating conventional dendritic cells, innate antigen presenting cells that potently activate naïve T cells, thereby bridging innate and adaptive immune responses.

**Methodology/Principal Findings:**

When challenged with LPS, Pin1-null mice failed to accumulate spleen conventional dendritic cells (cDC). Analysis of steady-state spleen DC populations revealed that Pin1-null mice had fewer CD8+ cDC. This defect was recapitulated by culturing Pin1-null bone marrow with the DC-instructive cytokine Flt3 Ligand. Additionally, injection of Flt3 Ligand for 9 days failed to induce robust expansion of CD8+ cDC in Pin1-null mice. Upon infection with *Listeria monocytogenes*, Pin1-null mice were defective in stimulating proliferation of adoptively transferred WT CD8+ T cells, suggesting that decreases in Pin1 null CD8+ cDC may affect T cell responses to infection *in vivo*. Finally, upon analyzing expression of proteins involved in DC development, elevated expression of PU.1 was detected in Pin1-null cells, which resulted from an increase in PU.1 protein half-life.

**Conclusions/Significance:**

We have identified a novel role for Pin1 as a modulator of CD8+ cDC development. Consistent with reduced numbers of CD8+ cDC in Pin1-null mice, we find that the absence of Pin1 impairs CD8+ T cell proliferation in response to infection with *Listeria monocytogenes*. These data suggest that, via regulation of CD8+ cDC production, Pin1 may serve as an important modulator of adaptive immunity.

## Introduction

Pin1 is a ubiquitously expressed phosphorylation-specific peptidyl prolyl isomerase (PPIase) that regulates substrate function by catalyzing the *cis-trans* isomerization of prolyl bonds. The ability of Pin1 to accelerate this intrinsically slow conformational switch enables it to function as a molecular timer whose activity appears to be crucial during events that require rapid or precisely timed responses [1], [2]. Consistent with such a role, Pin1 has been demonstrated to modulate the timing of primordial germ cell (PGC) proliferation in mice. In the absence of Pin1, PGC exhibit a prolonged cell cycle and undergo fewer cell divisions during their 5 day transit toward the fetal gonad. As a result, both male and female Pin1-null mice are born with fewer germ cells and display severe infertility [3]. Additional studies carried out in mouse embryo fibroblasts (MEF) indicate that the loss of Pin1 both delays entry into the cell cycle from quiescence and causes asynchronously growing cells to stall in the G1/S phase of the cell cycle [4], [5], [6].

Although multiple mammalian PPIases exist, Pin1 is unique in its ability to regulate substrates that are phosphorylated. By binding to phospho-Ser/Thr-Pro motifs, Pin1 has been shown to influence the dephosphorylation, stability, dimerization, localization, and/or activity of a diverse set of proteins [1], [7]. In addition to regulating both proliferation and survival in non-hematopoietic cells, Pin1 also possesses roles in modulating immune cell function. In collaboration with the Dalla-Favera laboratory, we previously showed that Pin1 regulates Bcl6 expression in germinal center B cells in response to genotoxic stress [8]. Pin1 also participates in the activation of T cells by modulating the activity of the transcription factor NFAT and regulating activation-induced cytokine production [9], [10]. Within the innate immune system, Pin1 has been shown to modulate allergen-induced cytokine production and survival in lung eosinophils [11], [12], [13]. Pin1 also modulates the turnover of the transcription factor IRF3 downstream of toll-like receptor (TLR) 3, and Pin1-null mice were defective in producing IFNβ when challenged with poly (I:C) to mimic viral infection [14]. A role for Pin1 has also been described in regulating endotoxemia and IL-6 mRNA production by activated macrophages [15]. Most recently, Pin1 was demonstrated to facilitate the production of IFNα in plasmacytoid dendritic cells via regulation of IRAK1 activity [16]. It is clear from these reports that Pin1 possesses the ability to regulate multiple arms of the immune response. Thus far, however, no role for Pin1 has been described in the most potent initiators of adaptive immunity, conventional dendritic cells.

Dendritic cells (DC) are innate antigen presenting cells that are particularly adept at activating naïve T cells and inducing immunologic memory [17]. Multiple DC subsets have been identified and differ in tissue distribution, receptor expression, and function. Conventional DC (cDC) and plasmacytoid DC (pDC) are two subsets that reside in lymphoid organs in close proximity to T cells [18]. cDC express multiple TLRs, which enable them to sense and respond to a variety of pathogens, including bacteria and virus. These cells are further divided into functional subsets based on expression of CD8. Those that lack CD8 expression are most abundant, and thought to primarily activate CD4+ T helper cell responses. CD8+ cDC are less abundant than CD8− cDC, and possess the ability to cross-present exogenous antigens to activate CD8+ T cells [17], [19], [20]. pDC express TLR7 and TLR9, which endow them with the ability to respond to viral nucleic acids. During viral infection, activated pDC support cDC and T cell function by secreting IFNα/β and T cell chemokines [21].

Dendritic cells develop from both common myeloid and lymphoid progenitors in the bone marrow, both of which can give rise to the common DC progenitor (CDP) [22]. This developmental program is dependent on the cytokine Flt3 Ligand (FL), which binds and activates the Flt3 receptor on hematopoietic progenitors. The requirement for this cytokine in DC development has been demonstrated in mice that lack either FL or Flt3 receptor, both of which exhibit profound defects in the production of cDC and pDC [23], [24]. Furthermore, administration of FL *in vivo* has been shown to induce massive expansion of DC in mice [25]. Efforts aimed at identifying molecular determinants of DC development and subset specification are ongoing. Many transcription factors have been identified that broadly regulate the development of multiple DC subsets, such as Stat3, which lies downstream of the Flt3 receptor [26]. Other transcriptional regulators appear to be more specific, such as Id2, which is reported to facilitate CD8+ cDC development and inhibit pDC development [27]. More recently, both NFIL3 and Batf3 have been shown to modulate the development of the CD8+ subset of cDC [19], [28]. Because the distinct functions of each DC subset shape and fine-tune the immune response [29], it is of great interest to identify specific modulators of subset development and function.

In this report, we describe a novel role for Pin1 in modulating the development of the CD8+ subset of cDC. Pin1-null mice have fewer steady-state CD8+ cDC in their spleens and are impaired in their ability to expand this subset *in vivo* in response to FL injection. These defects are not the result of decreased DC progenitors in the bone marrow, as Pin1-null bone marrow is comparable to that of WT mice. However, when Pin1-null bone marrow is cultured *ex vivo* with FL, it is defective in generating the CD8+ cDC equivalent subset. Furthermore, when infected with *Listeria monocytogenes* (*L.m.*), Pin1-null mice exhibit a reduced ability to induce expansion of adoptively transferred CD8+ T cells. Upon measuring the expression of transcription factors that regulate DC development, Pin1-null cells exhibited an increase in PU.1 protein expression, which results, in part, from decreased protein turnover. Thus, we propose Pin1 to be an important regulator of CD8+ cDC-dependent immune responses through its preferential modulation of CD8+ cDC development.

## Results

### Pin1-null mice fail to accumulate spleen cDC in response to LPS

**Figure 1:**
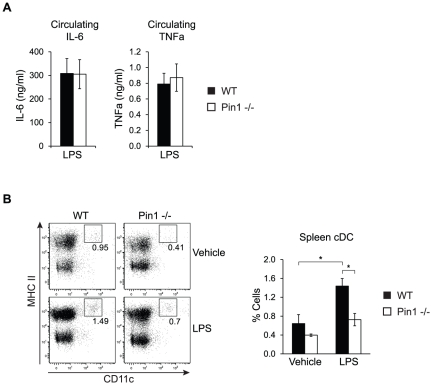
Pin1-null mice fail to accumulate spleen cDC after LPS challenge. (A) Circulating IL-6 and TNFα concentrations in WT and Pin1-null mice 3 hours after administration of 15 mg/kg LPS. Blood was collected either by tail vein bleed, or by cardiac puncture following euthanization. Samples were analyzed by ELISA (n = 6). (B) Representative FACS plots and graph of spleen cDC from WT and Pin1-null mice 18 hours after administration of either 15 mg/kg LPS or vehicle (HBSS). Graph shows the frequency of spleen cDC from WT and Pin1-null mice as percentage of total splenocytes (n = 6).

Pin1 has previously been described to modulate activation and cytokine production in both eosinophils and T cells [9], [11]. Based on these reports, we initially hypothesized that Pin1-null mice would exhibit an impaired response to systemic inflammation, which is characterized by activation of both innate antigen presenting cells and lymphocytes [30], [31]. Systemic inflammation was induced in mice by injecting the bacterial cell wall component lipopolysaccharide (LPS), as this is a well-established method to induce a sterile inflammatory response [32]. Three hours after LPS injection, blood was collected for measurement of two classic pro-inflammatory cytokines, IL-6 and TNFα. Pin1-null mice produced the same amounts of circulating IL-6 and TNFα as WT mice (Figure 1A).

Next, the presence of B cells, T cells and DC was determined by staining splenocytes with markers that identified these cell types. Eighteen hours after LPS injection, total B cells and T cells were comparable between WT and Pin1-null mice, as well as the LPS-stimulated expression of the activation marker CD69 (Figure S1). In contrast, Pin1-null mice that received LPS exhibited a significant defect in the accumulation of spleen cDC, which are identified by high expression of both CD11c and MHC class II (Figure 1B, S1C). Additionally, it appeared that vehicle-injected Pin1-null mice also had fewer cDC than WT mice, although this is only a slight trend. As Pin1 has not yet been ascribed any roles in cDC biology, the possibility that Pin1 could modulate steady-state cDC production and/or LPS-induced cDC accumulation was further investigated.

### Impaired production of steady-state spleen DC in Pin1-null mice

**Figure 2:**
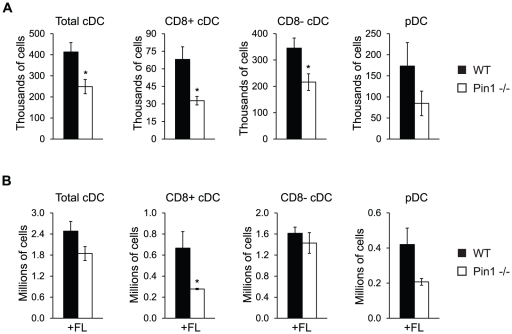
Pin1-null mice have fewer steady-state spleen cDC and fewer FL-expanded CD8+ cDC *in vivo*. (A) Quantitation of the numbers of steady-state spleen DC subsets from healthy WT and Pin1-null mice. Cell population numbers were determined by multiplying the frequency of the cell population by the total number of splenocytes obtained from each mouse (n = 6). Definitions of each cell population can be found in Materials and Methods. (B) Quantitation of the numbers of spleen DC subsets from WT and Pin1-null mice that were administered 1 µg Flt3 Ligand (FL) for 9 consecutive days. Cell population numbers were determined by multiplying the frequency of the cell population by the total number of splenocytes obtained from each mouse (n = 5).

To determine whether Pin1-null mice possess fewer steady-state spleen cDC, spleens were harvested from healthy WT and Pin1-null mice and stained for multiple DC populations. Pin1-null mice harbored a significant decrease in the number of both the CD8+ and CD8− subsets of spleen cDC, with the greatest defect in the CD8+ subset, which is decreased 50% compared to WT cells (Figure 2A). Upon examining the frequency of these populations, however, we encountered a slightly different result. While the frequency of Pin1-null CD8+ cDC remained significantly decreased compared to WT cells, there was not a significant decrease in the frequency of Pin1-null CD8− cDC (Figure S2A, S2B). The discrepancy between total number and frequency of CD8− cDC may be explained by the observation that Pin1-null mice tend to have fewer splenocytes than WT mice (Figure S2C). Although this trend does not reach statistical significance, when coupled to a trend for decreased frequency, it produces a significantly different total number. Pin1-null mice also exhibited a decrease in both the number and frequency pDC but neither of these differences was statistically significant (Figure 2A, S2A, S2B). Despite our uncertainty regarding the existence of a defect in Pin1-null CD8− cDC, the data clearly indicated that the absence of Pin1 disrupts the ability of CD8+ cDC to populate the spleen under steady-state conditions.

We next examined a potential role for Pin1 in cDC development by injecting mice with FL and measuring the resulting expansion of DC subsets. Mice were injected with 1 µg of FL for 9 consecutive days, as has previously been described [33]. On day 10, splenocytes were stained and DC populations were quantified. Pin1-null mice were unable to expand the CD8+ subset of cDC to the same extent as WT mice. The FL-induced accumulation of CD8− cDC, however, was comparable between WT and Pin1-null mice. This result is consistent with the absence of a decrease in the frequency of steady-state CD8− cDC in Pin1-null mice (Figure S2A). Similar to what was observed in the steady-state, FL-treated Pin1-null mice accumulated fewer pDC, but again this difference does not reach statistical significance (Figure 2B). Taken together, these results suggest that CD8+ cDC are particularly sensitive to the loss of Pin1, as they exhibit the greatest defect in its absence during both steady-state conditions and FL-induced expansion *in vivo*.

### Bone marrow progenitors are unaltered in the absence of Pin1

**Figure 3:**
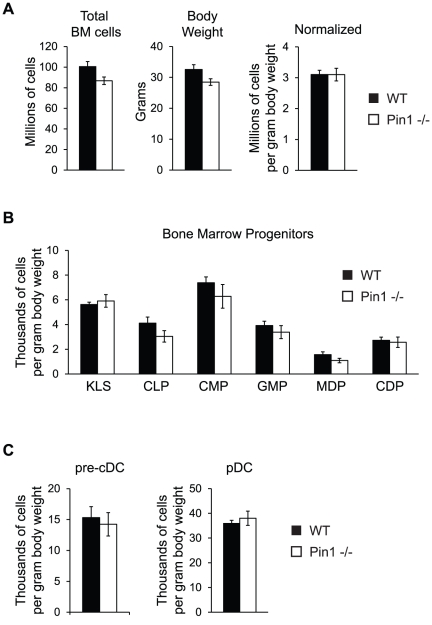
DC bone marrow progenitors are unaltered in absence of Pin1. (A) Normalization of total bone marrow cells from WT and Pin1-null mice by weight (grams). WT and Pin1-null mice were weighed upon euthanization. The normalized graph was generated by dividing the total number of bone marrow cells by the body weight (grams) of each mouse (n = 6). (B) Quantitation of normalized numbers of bone marrow progenitors in both WT and Pin1-null mice. KLS, c-kit+Lin-sca1+ stem cells; CLP, common lymphoid progenitor; CMP, common myeloid progenitor; GMP, granulocyte-macrophage progenitor; MDP, macrophage-dendritic cell progenitor; CDP, common dendritic cell progenitor. Total cell numbers were determined by multiplying the frequency of each population by the total number of bone marrow cells. Total population numbers were then normalized to body weight by dividing the number of cells in each population by the body weight (grams) of each mouse (n = 6). (C) Quantitation of normalized numbers of pre-cDC and pDC in the bone marrow of WT and Pin1-null mice. Normalized cell numbers were calculated as described in (B) (n = 6).

DC develop from hematopoietic progenitors in the bone marrow that transition through several stages of development, becoming increasingly committed to one particular fate with each subsequent step. To address whether defects existed in bone marrow progenitors of Pin1-null mice that could account for the changes observed in the spleen DC populations, bone marrow cells from WT and Pin1-null mice were stained and analyzed for the presence of multiple progenitors (schematic of DC development available in Figure S3). As noted with the number of splenocytes, Pin1-null mice exhibited a trend for reduced numbers of bone marrow cells. When corrected for differences in total body weight, however, these differences no longer existed (Figure 3A). Upon normalizing by body weight, no defects in the number of Pin1-null bone marrow progenitors were detected (Figure 3B). These results are consistent with the frequencies of bone marrow progenitors, which are also unaltered in Pin1-null mice (Figure S4A).

pDC fully develop within the bone marrow, while pre-cDC leave the bone marrow and circulate to peripheral tissues where they undergo the final steps of development to give rise to CD8− or CD8+ cDC (Figure S3). To determine whether defects existed in these two populations, bone marrow cells were also stained with markers of pre-cDC and pDC. Consistent with an absence of defects in bone marrow progenitors, neither of these populations was perturbed in Pin1-null mice, either in number (Figure 3C) or frequency (Figure S4B). The absence of a defect in Pin1-null bone marrow pDC is interesting in light of the trend to have fewer spleen pDC, and suggests that changes in spleen pDC number are not the result of impaired development, but may instead arise from a separate defect. Collectively, our data indicate that the loss of Pin1 is inconsequential to stages of cDC and pDC development that take place in the bone marrow, and point to a role for Pin1 in processes that occur in the periphery.

### Loss of Pin1 impairs the ability of cultured bone marrow to generate cDC

**Figure 4:**
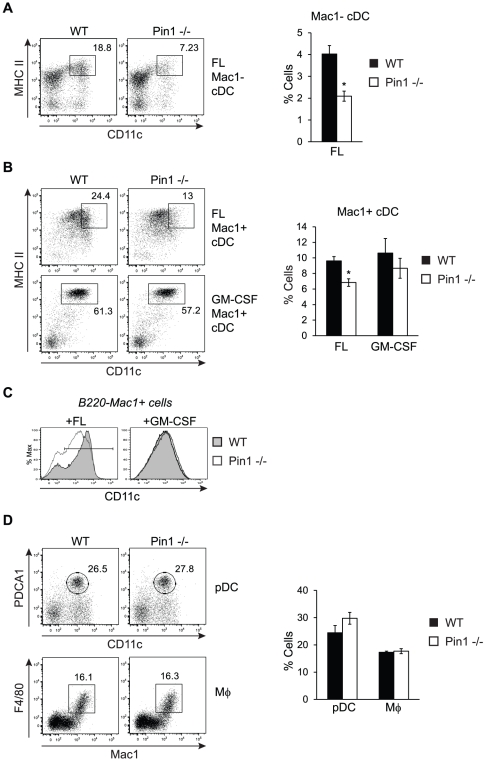
DC development in bone marrow cultures. (A) Representative FACS plots of WT and Pin1-null bone marrow-derived Mac1- cDC generated by culturing with FL. Cells were previously gated as B220-Mac1− (“Mac1−”) and cDC are identified as MHCII^hi^CD11c^hi^ cells. The frequency of Mac1− cDC corresponding to the populations gated in the FACS plots on the left are graphed on the right as percentage of total cells (n = 16). (B) Representative FACS plots of WT and Pin1-null bone marrow-derived Mac1+ cDC generated by culturing with either FL (n = 16) or GM-CSF (n = 11). FL-generated cells were previously gated as B220-Mac1+, and GM-CSF-generated cells were previously gated as B220-Mac1+GR1-. In the plots shown, cDC are identified as MHCII^hi^CD11c^hi^ cells. The frequencies of Mac1+ cDC gated on the left are depicted in the graph on the right as the percentage of total cells. (C) Representative overlaid histograms comparing CD11c expression in WT and Pin1-null B220-Mac1+ cells generated from FL and GM-CSF bone marrow cultures. (D) Representative FACS plots of WT and Pin1-null bone marrow-derived pDC and macrophage (mΦ). Cells were not previously gated. pDC are identified as PDCA1+CD11c^int^ cells and macrophages are identified as F4/80+Mac1+ cells. The frequencies of WT and Pin1-null bone marrow-derived pDC (n = 16) and macrophages (n = 6) are shown in the graph on the right as percentage of total cells.

To determine if Pin1 regulates final stages of CD8+ cDC development that occur outside the bone marrow, and to eliminate the potential contribution of altered migration to the spleen, an *ex vivo* bone marrow culture system was utilized to induce DC development. WT and Pin1-null bone marrow cells were cultured in the presence of FL for 9 days, an established regimen that generates fully developed pDC and cDC subsets that are functionally equivalent to steady-state populations *in vivo*. Although bone marrow-derived cDC do not express CD8, the two subsets have previously been distinguished from each other by the presence or absence of the myeloid marker Mac1 [34], [35]. When cultured with FL, Pin1-null bone marrow exhibited a 50% reduction in the generation of Mac1- (CD8+ equivalent) cDC, which mirrored what had been observed *in vivo* (Figure 4A). The Mac1+ (CD8− equivalent) subset, however, exhibited a more complex phenotype in the absence of Pin1. Rather than being reduced in number, FL-cultured Pin1-null Mac1+ cDC appear to express less CD11c than WT Mac1+ cDC. Indeed, when gated on the brightest CD11c+ cells, a significant decrease in bone marrow-derived Mac1+ cDC can be quantified (Figures 4B, 4C). Because a similar decrease in CD11c expression was not observed in CD8− cDC *in vivo* (data not shown), the significance of this observation remained uncertain. To further examine Mac1+ (CD8− equivalent) cDC development, we cultured bone marrow cells with the cytokine GM-CSF, which has previously been shown to exclusively generate CD8− cDC equivalents, but not CD8+ cDC equivalents or pDC [34], [36]. Under these culture conditions, we did not observe a defect in the production of Pin1-null Mac1+ cDC, nor did we detect differences in the expression of CD11c (Figures 4B, 4C). Based on our collective results, we conclude that Pin1 is unlikely to have a substantial role in the development of the CD8− subset of cDC.

Consistent with results obtained from freshly isolated bone marrow cells, the generation of pDC was unaltered in Pin1-null bone marrow cultures, further indicating that Pin1 is dispensable for pDC development (Figure 4D). To further investigate the possibility that the absence of Pin1 imparts a general developmental deficiency on bone marrow progenitors, Pin1-null bone marrow was also assayed for its ability to produce macrophages, cells that share common myeloid progenitors with DC and similarly undergo the final stages of development in peripheral tissues (Figure S3) [37]. After 6 days in culture with the cytokine macrophage colony stimulating factor (M-CSF), both adherent and non-adherent cells were collected and stained. No defect was found in the generation of macrophages from Pin1-null bone marrow cultures (Figure 4D). Taken together, the absence of defects in the generation of both pDC and macrophages in Pin1-null bone marrow cultures indicates that Pin1 may be preferentially required for late stages of cDC development.

**Figure 5:**
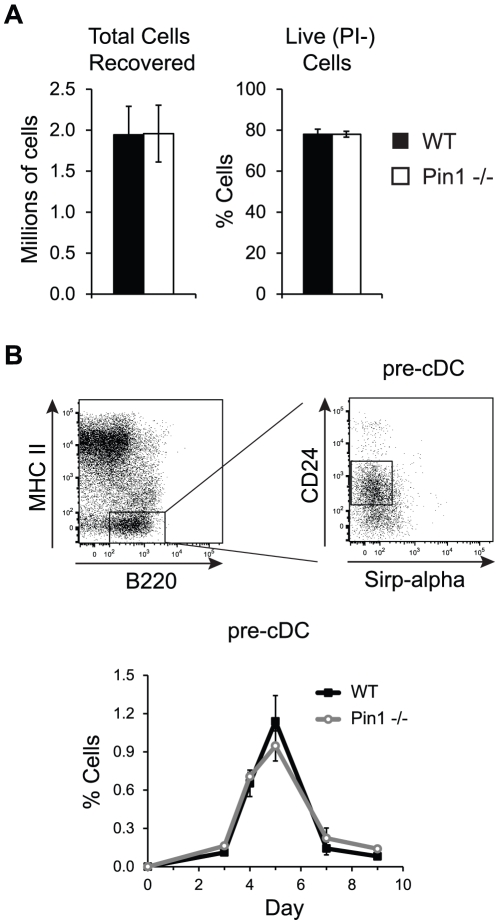
Cell survival and pre-cDC generation in bone marrow cultures. (A) The graph on the left shows the quantitation of the total number of WT and Pin1-null bone marrow-derived dendritic cells recovered from bone marrow cultures after 9 days incubation with FL. The graph on the right is a quantitation of the frequency of live WT and Pin1-null bone marrow-derived DC that did not stain with propidium iodide (PI−) (n = 3). (B) The top panel depicts part of the gating strategy used to identify pre-cDC. Previously gated PDCA1-CD11c+ cells were further gated, as shown, to identify PDCA1-CD11c+MHCII-B220+ Sirpα-CD24+ pre-cDC in FL cultured bone marrow cells. The frequencies of pre-cDC from WT and Pin1-null cultures are quantified in the graph below as percentage of total cells (n = 3).

To determine whether the decrease in Mac1- (CD8+ equivalent) cDC observed in Pin1-null cultures resulted from decreased proliferation or survival, we compared total cell numbers and propidium iodide (PI) uptake between WT and Pin1-null cells. We did not detect any differences in the total number of cells recovered on day 9 of culture, nor did we observe any dissimilarities in the frequency of live (PI−) cells (Figure 5A). These results indicate that the decrease in Pin1-null Mac1- cells is unlikely to be due to altered proliferation or survival. Another possible explanation for decreased Pin1-null Mac1- cDC is an impairment in the production of the immediate precursor to Mac1- cDC. A population of pre-cDC that preferentially gives rise to both the CD8+ cDC equivalent and CD103+ DC, but not CD8− cDC, has recently been described in FL bone marrow cultures [38]. Although the authors utilized transgenic cells expressing GFP-Id2 to identify this population, the cells were further characterized as CD11c+MHCII-B220+PDCA1-Sirpα-CD24+. We collected WT and Pin1-null cells between days 3 and 9 of culture with FL and determined the frequency of CD11c+MHCII-B220+PDCA1-Sirpα-CD24+ cells. We were unable to detect differences in this population between WT and Pin1-null cultures on any of the days assessed (Figure 4B). It is possible that without gating on Id2-expressing cells we were unable to accurately identify this subset of pre-cDC. Additionally, we cannot exclude the possibility that there exists a defect in another population of Pin1-null pre-CD8+ cDC that has not yet been identified.

Similar to Mac1 expression, the expression of Sirpα and CD24 have been used to distinguish between CD8+ and CD8− subsets of cDC both *in vivo* and in *ex vivo* FL cultures [38], [39]. To confirm our previous results, we used these additional markers to identify the fully developed B220-MHCII+CD24+Sirpα− (CD8+ equivalent) and B220-MHCII+CD24(lo)Sirpα+ (CD8− equivalent) cDC populations. Consistent with results obtained by gating on Mac1 expression, Pin1-null FL cultures exhibited a significant decrease in the CD24+Sirpα− (CD8+ equivalent) cDC population of cells compared to WT cultures. Furthermore, the CD24(lo)Sirpα+ (CD8− equivalent) cDC population was not significantly altered in the absence of Pin1 (Figure S5). The ability of *ex vivo* bone marrow cultures to recapitulate the reduced number of CD8+ cDC observed in Pin1-null spleens suggests that Pin1 functions as a modulator of late CD8+ cDC development.

### Pin1 is dispensable for LPS-induced MHC class II and co-stimulatory molecule expression

**Figure 6:**
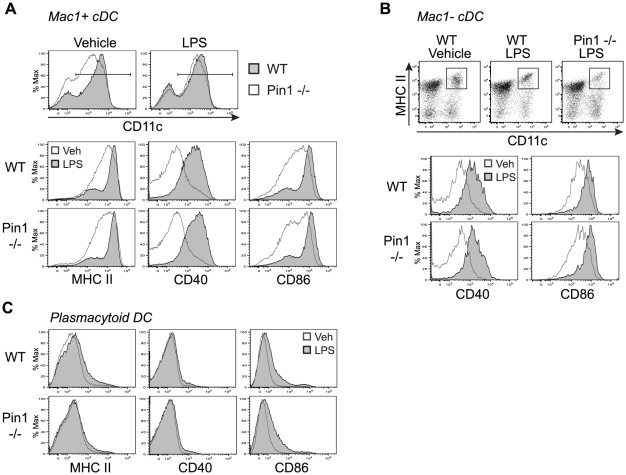
Pin1-null bone marrow-derived DC up-regulate costimulatory molecule expression in response to LPS. (A) Representative histograms showing expression of MHC class II (MHCII) and costimulatory molecules in WT and Pin1-null Mac1+ bone marrow-derived DC that were unstimulated or stimulated with 100 ng/ml LPS for 24 hours. The upper panel shows a histogram of CD11c expression in previously gated B220-Mac1+ cells. The gate shown in the histogram was applied to further analyze all CD11c+ cells for the expression of MHC class II (MHCII), CD40, and CD86 in the lower panels. Histograms represent data acquired from two independent experiments (n = 6). (B) Representative histograms showing expression costimulatory molecules in WT and Pin1-null Mac1− bone marrow-derived DC that were unstimulated or stimulated with 100 ng/ml LPS for 24 hours. The upper panel shows FACS plots of Mac1− cDC that were previously gated as B220-Mac1−. The gate in the upper panel (MHCII+CD11c+ gate) was applied to further analyze Mac1− cDC for the expression of CD40 and CD86 in the lower panels. Histograms represent data acquired from two independent experiments (n = 6). (C) Representative histograms showing expression of MHC class II (MHCII) and costimulatory molecules in WT and Pin1-null bone marrow-derived pDC that were unstimulated or stimulated with 100 ng/ml LPS for 24 hours. pDC were previously gated as PDCA1+CD11c+. Histograms represent data acquired from two independent experiments (n = 6).

When cDC encounter foreign antigen they become activated and undergo maturation, a process that endows them with the ability to stimulate T cells. To determine whether the loss of Pin1 alters cDC maturation, day 9 FL bone marrow cultures were incubated with LPS for 24 hours and analyzed for the expression of MHC class II and the co-stimulatory molecules CD40 and CD86, which are induced upon maturation. Upon LPS stimulation, the defect in CD11c expression present in unstimulated Pin1-null Mac1+ cDC became undetectable; both WT and Pin1-null cells expressed CD11c to the same extent (Figure 6A). While the reason for decreased CD11c expression in FL cultures remains unclear, it does not appear that this defect extends to mature Mac1+ cDC, and is therefore unlikely to directly impact the ability of DC to stimulate adaptive immune responses.

When analyzed for the expression of MHC class II, CD40 and CD86, both Mac1- and Mac1+ subsets of Pin1-null cDC expressed each of these molecules to the same extent as WT cells when stimulated with LPS (Figure 6A, 6B). Neither WT nor Pin1-null pDC increased expression of these proteins, as was expected since pDC lack TLR4 and are unable to respond to LPS (Figure 6C). Thus, although there are fewer Pin1-null Mac1- cDC, those cells that develop are capable of up-regulating MHC class II and co-stimulatory molecule expression in response to LPS.

### CD8+ T cell response in Pin1-null mice infected with *Listeria monocytogenes*

**Figure 7:**
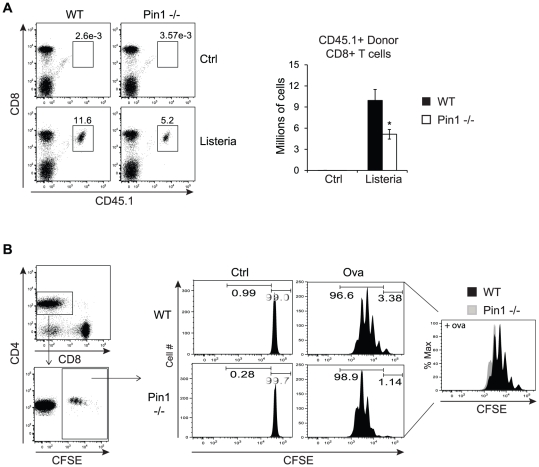
Impaired CD8+ T cell proliferation in Pin1-null mice infected with *Listeria monocytogenes*. (A) Representative FACS plots of donor WT CD45.1+CD8+ OT1 T cells isolated from spleens of CD45.2+ WT and Pin1-null mice that were previously injected with 10,000 WT CD8+CD45.1+ ova-specific T cells and then either left uninfected or infected with *L.m.*-ova for 7 days. Cells were previously gated as CD3+. The graph on the right is a quantitation of WT CD45.1+CD8+ OT1 T cells obtained from the spleens of WT and Pin1-null mice. Total numbers of CD8+ CD45.1+ cells were determined by multiplying the cell frequency by the total number of splenocytes obtained from each mouse (n = 4). (B) The plots on the left show the previous gates used to identify donor WT CFSE+ CD4+ OT2 T cells isolated from spleens of WT and Pin1-null mice that were previously injected with 1–2 million WT CSFE-labeled ova-specific CD4+ T cells. The four panels in the center are representative histograms (n = 3) showing CFSE intensity in WT and Pin1-null mice that were either unchallenged (ctrl) or injected with ova peptide (ova). The overlaid histogram on the right is an additional depiction of the two ova histograms to directly compare CFSE intensity between CD4+ T cells derived from WT and Pin1-null mice.

The most robust defect observed in Pin1-null mice and Pin1-null bone marrow cultures was impaired generation of CD8+ cDC. To test the hypothesis that decreased CD8+ cDC in Pin1-null mice would impact the proliferation of CD8+ T cells *in vivo*, mice were infected with *Listeria monocytogenes (L.m.)*, an intracellular bacterium that has been demonstrated to both activate CD8+ cDC and induce CD8+ T cell priming [40], [41], [42]. To eliminate the possibility that a detectable difference in T cell expansion might result from a T cell-intrinsic defect in the absence of Pin1, WT and Pin1-null mice were injected with WT transgenic CD8+ T cells from OT1 mice. These cells express an ovalbumin(ova)-specific T cell receptor as well as the CD45 allelic variant CD45.1, which allowed them to be distinguished from endogenous CD45.2-expressing cells. Twenty-four hours after receiving 10,000 OT1 CD8+ T cells, WT and Pin1-null mice were administered 1×10^4^ CFU of live *L.m.* engineered to express ovalbumin, thereby enabling the induction of a strong response from the ova-specific OT1 CD8+ T cells. Seven days after *L.m.* infection, mice were euthanized and splenocytes were stained for CD45.1+ CD8+ T cells. Pin1-null mice accumulated 50% fewer CD45.1+ CD8+ T cells than WT mice (Figure 7A).

To determine the specificity of defective CD8+ T cell proliferation in Pin1-null mice, we also examined the proliferation of adoptively transferred WT CD4+ T cells. Both WT and Pin1-null mice were injected with 1–2 million CFSE-labeled WT CD4+ T cells from OT2 mice, which also express an ova-specific T cell receptor. Twenty-four hours later, mice were injected with ova peptide (323–339) to stimulate proliferation of ova-specific CD4+ T cells. After an additional 48 hours, splenocytes were collected and stained to identify CFSE+ CD4+ T cells. A single peak corresponding to CFSE bright CD4+ T cells was detected in both WT and Pin1-null control mice that did not receive ova peptide. As expected, the CD4+ T cells of mice that received ova exhibited multiple peaks of decreasing CFSE intensity, which correspond to successive rounds of cell division. No defects were observed in the proliferation of adoptively transferred CD4+ OT2 cells in Pin1-null mice. Rather, it appeared that CD4+ T cell proliferation was mildly enhanced in the absence of Pin1, as there was a slight shift towards more CFSE dilute CD4+ T cells in Pin1-null mice (Figure 7B). Taken together, these results are consistent with impaired CD8+ cDC development in Pin1-null mice and suggest that such a defect has the ability to impair CD8+ T cell proliferation *in vivo*.

### Pin1 regulates expression of PU.1

**Figure 8:**
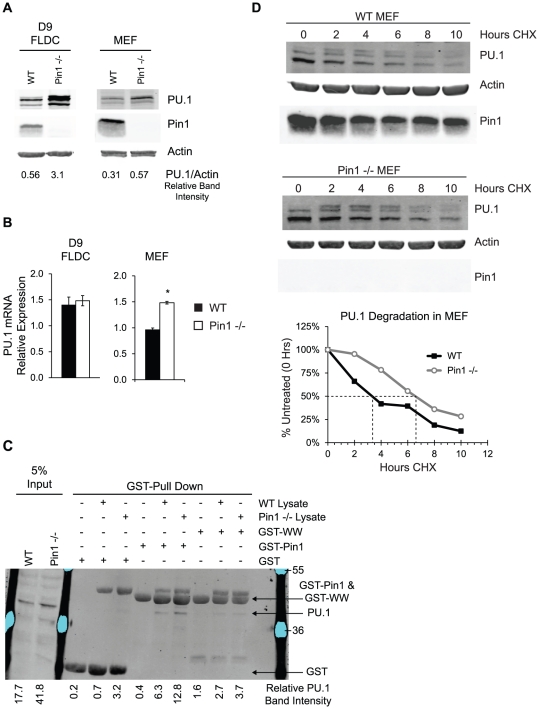
Pin1 modulates PU.1 protein stability. (A) Immunoblot analysis of PU.1 protein expression in WT and Pin1-null FL-cultured bone marrow-derived DC (FLDC) and primary MEF. For FLDC, the immunoblot shown is representative of cells derived from 3 different mice. For MEF, the immunoblot shown is representative of 3 different experiments. (B) Quantitation of PU.1 mRNA expression from WT and Pin1-null FLDC (n = 5) and MEF (n = 3). (C) GST-Pin1 pull down in WT and Pin1-null MEF lysates. 1 mg of total lysate was incubated with GST alone, WT GST-Pin1, or WW GST-Pin1 for 2 hours. After binding, beads were washed, resuspended in SDS-Page sample buffer, boiled, and then analyzed by immunoblot. Membranes were probed for expression of PU.1. (D) PU.1 protein expression in WT and Pin1-null MEFs after being treated with 150 µg/ml cycloheximide (CHX) to inhibit protein synthesis for 2, 4, 6, 8, or 10 hours. The immunoblot shown is representative of two independent experiments. PU.1 protein expression is plotted in the bottom graph as a percentage of total PU.1 protein at time zero, and reflects the values obtained from immunoblots shown directly above.

To further understand how Pin1 modulates cDC development, immunoblot analysis was performed on lysates from WT and Pin1-null FL-generated bone marrow DC (FLDC). The expression of several different proteins previously identified as regulators of cDC development was measured, including IRF8, Id2, Gfi-1, and PU.1 [33], [43], [44]. Although the expression of the other proteins appeared to be unaltered (Figure S6), there was a marked increase in PU.1 protein in Pin1-null FLDC. This same deregulation was confirmed in Pin1-null mouse embryo fibroblasts (MEF), which also express elevated levels of PU.1 protein (Figure 8A). To determine whether increased PU.1 protein was the result of elevated PU.1 mRNA, qRT-PCR analysis was performed on both FLDC and MEF. No change in PU.1 mRNA was detected in Pin1-null FLDC. qRT-PCR analysis in MEF, however, revealed a modest 1.5-fold increase in PU.1 mRNA in the absence of Pin1 (Figure 8B). As PU.1 is known to bind and regulate its own promoter, we speculate that increased PU.1 mRNA in Pin1-null MEF might result from enhancement of this feed-forward loop. The absence of the same increase in PU.1 mRNA in FLDC, however, may be indicative of additional defects in PU.1 transcriptional activity. Indeed, PU.1 is known to interact with multiple proteins that co-regulate transcription of target genes [45]. Because several of these PU.1-binding proteins are absent in MEF (Figure S6), it is possible that PU.1 transcriptional activity is not regulated in the same manner in MEF as in FLDC.

It was recently published that GST-Pin1 binds endogenous PU.1 in PMA-stimulated THP-1 human macrophages [15]. This interaction was confirmed by performing a GST pull-down assay in both WT and Pin1-null MEF and showing that endogenous PU.1 is able to bind WT GST-Pin1. The inability of a WW binding domain mutant of GST-Pin1 (W11A, W34A) to bind PU.1 indicated that this domain of Pin1 is necessary to maintain the interaction with PU.1 (Figure 8C), as was previously reported in COS-7 cells [15]. Three putative Pin1 binding sites exist in the PU.1 protein, two of which are located in the PEST domain. Because PEST domains have been shown to modulate the degradation of other proteins, the ability of Pin1 to regulate PU.1 protein turnover was examined [46]. Both WT and Pin1-null MEF were incubated with the protein synthesis inhibitor cycloheximide and the rate of PU.1 degradation was assessed by immunoblot analysis. The absence of Pin1 was found to promote PU.1 stability, nearly doubling its half-life (Figure 8D). These data further expand on the recently-described interaction between Pin1 and PU.1 by providing new evidence that Pin1 facilitates PU.1 protein degradation.

## Discussion

This work was begun to identify a role for Pin1 in modulating systemic inflammation, as such a role had not yet been described. Three hours after administration of LPS, Pin1-null mice did not exhibit defects in circulating levels of two classic pro-inflammatory cytokines, IL-6 and TNFα. This result initially seems to contradict the recent claim that Pin1 regulates serum IL-6 production in a model of endotoxic shock [15]. In that report, however, measurements were taken 24 hours after challenge, a time point at which circulating levels of cytokines have previously been shown to be in decline [47], [48]. For this reason, we do not believe that our results are directly comparable to those data previously described. Upon staining splenocytes, an impairment in the accumulation of spleen cDC was identified in Pin1-null mice. Although 18 hours after challenge is not a typical time to measure cDC accumulation, we nonetheless observed a defect that suggested to us that Pin1 may play some role in cDC biology, which has not been described. Indeed, further investigation into this unexpected result uncovered a novel role for Pin1 in regulating steady-state production of CD8+ cDC. We observed significant decreases in both the total number and frequency of steady-state CD8+ spleen cDC in Pin1-null mice. Additionally, Pin1-null mice were significantly impaired in their ability to accumulate CD8+ cDC, but not CD8− cDC or pDC, in response to *in vivo* administration of FL.

In order to determine whether the observed defect in spleen CD8+ cDC accumulation resulted from defects in Pin1-null bone marrow cells, DC progenitors and pre-cDC were quantified in the bone marrow of WT and Pin1-null mice. No defects in progenitors or pre-cDC were observed in the bone marrow of Pin1-null mice, indicating that Pin1 is not required for early stages of cDC development. Similar results have been obtained in both Batf3-null mice and NFIL3-null mice, which also lack defects in bone marrow pre-cDC despite exhibiting impaired CD8+ cDC development [49], [50]. Because pDC fully develop within the bone marrow, they were also quantified. The absence of Pin1 did not alter numbers of bone marrow pDC, indicating that Pin1 is not required for the development of this subset.

Upon determining that early stages of cDC development were unperturbed in the bone marrow of Pin1-null mice, we utilized established *ex vivo* bone marrow culture protocols to examine later stages of cDC development. Because cDC in FL cultures do not express CD8, other cell surface markers have previously been used to distinguish between the CD8+ and CD8− cDC equivalents. We stained FLDC for two different sets of markers that have been described to specifically discriminate between the two cDC subsets. Both sets of markers clearly indicated a significant defect in the CD8+ cDC equivalent subset of cDC in Pin1-null cultures, which mirrored the defect identified in steady-state CD8+ cDC *in vivo*. Upon quantifying the CD8− cDC equivalent population in bone marrow cultures, we encountered slightly more complex results. When cultured with FL and gated on Mac1 expression, Pin1-null Mac1+ (CD8− equivalent) cDC expressed less CD11c than WT Mac1+ cDC. To help clarify this result, bone marrow was also cultured with GM-CSF to exclusively produce the Mac1+ (CD8− equivalent) subset of cDC. Under these conditions, Pin1-null Mac1+ cDC production was not impaired, nor was there any observable decrease in CD11c expression. Additionally, when FL cultured cells were alternately stained with CD24 and Sirpα, which have previously been used to discriminate between cDC subsets, we did not observe a decrease in the Pin1-null CD8− cDC equivalent cells, which are defined as CD24(lo)Sirpα+ cells. Taken together, these results indicate that Pin1 is unlikely to be required for the development of CD8− cDC. Conversely, Pin1 appears to modulate the development of CD8+ cDC, as indicated by the significant decrease in CD8+ equivalent cDC that was consistently observed in Pin1-null bone marrow cultures.

Coupling our observations in Pin1-null mice to the results from our *ex vivo* bone marrow culture system indicates a role for Pin1in preferentially regulating the development of the CD8+ subset of cDC. Few proteins have been described to regulate the development of one particular DC subset, as many are transcription factors that regulate early stages of development [51]. Although more restricted transcription factors have been identified, including IRF8 and Id2 [43], many of these proteins have been reported to possess additional roles in regulating the development and/or function of other hematopoietic lineages [44], [52], [53], [54], [55]. While we cannot rule out subtle defects in the development of other subsets of DC, Pin1 appears to be particularly important for the production of CD8+ cDC. We find this interesting since, compared to the CD8− subset of cDC, CD8+ cDC have been shown to exhibit more rapid BrdU labeling kinetics, indicating that these cells are produced and turned over more quickly than CD8− cDC [56], [57]. Additionally, under conditions that stimulate DC expansion *in vivo*, such as challenge with monophosphoryl lipid A, injection of FL, and bone marrow transplantation, the CD8+ subset of cDC has been demonstrated to exhibit the greatest degree of expansion [56], [58]. Accordingly, it is conceivable that delayed development in the absence of Pin1 could give rise to a more pronounced defect in the accumulation of the CD8+ subset of cDC, which is rapidly turned over *in vivo*. Such a scenario would be consistent with previously described roles for Pin1 as a rate-limiting modulator of precisely timed processes.

To address whether the observed defect in the production of Pin1-null CD8+ cDC can influence adaptive immune responses *in vivo*, we evaluated the effects of a pathogen that induces CD8+ cDC activation as well as CD8+ T cell priming. Acknowledging that Pin1 has already been shown to regulate the production of type I interferons in response to either poly (I:C) or virus [14], [16], we infected mice with *Listeria monocytogenes (L.m.)*, an intracellular bacterium that has been demonstrated to induce CD8+ T cell proliferation. *L.m.*-infected Pin1-null mice were found to be defective in their ability to expand adoptively transferred WT CD8+ T cells. Because CD8+ cDC have previously been shown to stimulate proliferation of CD8+ T cells [40], [41], these results are consistent with reduced production of CD8+ cDC observed in Pin1-null mice. Furthermore, these data support the idea that manipulation of Pin1 may be valuable for modulating CD8+ cDC-dependent immune responses *in vivo*.

To investigate how Pin1 modulates cDC development, the expression of several proteins reported to participate in DC development was determined. Immunoblot analysis revealed that Pin1-null FLDC and MEF expressed greater amounts of PU.1 protein than WT cells. When PU.1 mRNA levels were measured, there appeared to be a discrepancy between FLDC and MEF; PU.1 mRNA was unchanged in Pin1-null FLDC, but slightly elevated in MEF. This modest increase in PU.1 mRNA in MEF may be due to the ability of PU.1 to bind its own promoter and activate transcription [45]. As transcriptional activity appears to be cell-type dependent and regulated by coordinated interactions with other cell-specific proteins, it is possible that differences exist between FLDC and MEF in the regulation of PU.1 activity [59]. This hypothesis is supported by the fact that previously-described PU.1 binding proteins, such as IRF8 and Gfi-1, were undetectable in MEF [60], [61].

The abundance of PU.1 protein varies between different lineages and developmental stages, indicating that regulated changes in expression may be important, and perhaps instructive, for lineage-specific development of both myeloid and lymphoid cells [62], [63]. The role of PU.1 in DC development is not fully understood, and appears to be quite complex. Indeed, PU.1 can both positively and negatively regulate gene transcription, and its activity is influenced by interaction with other proteins as well as phosphorylation [45], [60], [61], [64], [65]. Two putative Pin1 binding sites are located within the PEST domain of PU.1, a region that has been shown to mediate interactions between PU.1 and other proteins [61]. Our results confirm the recent report that Pin1 binds to PU.1, and that this interaction is abolished upon mutation of the Pin1 WW domain [15]. Adding to the understanding of this relationship, Pin1 was determined to regulate PU.1 protein turnover, as indicated by the doubling of PU.1 protein half-life in the absence of Pin1. Modulating protein degradation is a common mechanism by which Pin1 regulates the activity of its substrates. Indeed, Pin1 has also been shown to regulate the stability and turnover of other hematopoietic transcription factors, including NF-κB p65, IRF3, and Bcl6 [8], [14], [66]. Although we do not provide direct evidence, it is tempting to speculate that Pin1 might regulate CD8+ cDC development through cell-specific modulation of PU.1 activity, which could be achieved by regulating PU.1 degradation rate, interactions with binding partners, and perhaps dephosphorylation, as has been shown for other Pin1 substrates [2], [67], [68]. Further work is required to understand how Pin1 binding to PU.1 is regulated, and how this interaction might impact PU.1 function.

In conclusion, we have expanded the current understanding of DC development by identifying a novel role for Pin1 in regulating the steady-state production of CD8+ cDC. The absence of Pin1 impairs FL-induced expansion of CD8+ cDC and also prevents robust proliferation of WT CD8+ T cells following bacterial infection in mice. Collectively, these results establish Pin1 as an important modulator of CD8+ cDC development, and further implicate Pin1 as a regulator of innate immunity.

## Materials and Methods

All animal work was conducted under Duke University Institutional Animal Care and Use Committee protocol numbers A073-09-03 (ARM) and A172-09-06 (YWH).

### Mice

CD45.2+ Pin1 −/− mice were generated as previously described and maintained in a pure C57BL/6 background along with WT littermates [3].

### Reagents

Lipopolysaccharide from *Escherichia coli*, Sigma L2637; Hanks Balanced Salt Solution (HBSS) without calcium, magnesium and phenol red, Mediatech, Inc. 21-022-CV; EDTA-coated Microtainers, BD Biosciences 365974; Serum separator tubes, BD Biosciences 365956; IL-6 ELISA Max kit, BioLegend 431302; TNFα ELISA Max kit, BioLegend 430902; Dulbecco's Modified Eagle's Medium (DMEM), Mediatech, Inc. 17-205-CV; heat-inactivated Fetal Bovine Serum (hiFBS), Gemini BioProducts 900-108; recombinant mouse Flt3 Ligand, R&D 427-FL/CF; recombinant mouse GM-CSF, eBioscience BMS325; recombinant mouse M-CSF, eBioscience 14-8983; 1× RBC Lysis Buffer, eBioscience 00-4333-57; CFDA-SE Cell Tracer Kit, Invitrogen V12883; ovalbumin (ova) peptide (323–339), AnaSpec 27025; Sigma Adjuvant System, Sigma-Aldrich S6322; Cycloheximide, Calbiochem 239763.

### Measurement of circulating cytokines by ELISA

Three hours after injection of LPS, blood was either obtained by tail vein bleed, or from cardiac puncture after euthanization. Tail vein blood was collected in EDTA-coated microtainers and then centrifuged at 5,000 rpm and 4°C for 15 minutes to pellet cells. Supernatant was collected (plasma) and cytokines were measured by ELISA. Blood from cardiac puncture was collected in a serum separator tube and centrifuged at 10,000 rpm for 10 minutes at room temperature to separate serum. Serum was removed from top fraction and cytokines were measured by ELISA.

### Isolation of splenocytes and bone marrow cells

To obtain splenocytes, spleens were removed and crushed on ice in DMEM-BM (DMEM supplemented with Pen/Strep, 10% hiFBS, 2 mM L-glutamine, 1 mM Sodium Pyruvate, MEM Non-essential Amino Acids, 10 mM HEPES, and 0.1% β-Mercaptoethanol). To obtain bone marrow cells, both femurs and tibias were removed and the marrow was flushed out of the bones with DMEM-BM using a needle and syringe. For both splenocytes and bone marrow cells, erythrocytes were removed by lysis in 1× RBC Lysis Buffer for 4–6 minutes on ice. Cells were then washed, passed through a 70 µm cell strainer, counted, and either directly stained or put into culture.

### Staining and flow cytometry

Between one and five million cells were washed in FACS Buffer (HBSS containing 3% hiFBS, 0.1% NaN_3_, and 10 mM EDTA) and then pelleted, resuspended in 100 ul Antibody Dilution Buffer (FACS Buffer supplemented with 5% Normal Mouse Serum, 5% Normal Rat Serum, and 1% purified anti-CD16/32(FcγR)), and incubated on ice for 15 minutes. Primary antibodies were then added to cells in Antibody Dilution Buffer (ADB) for 25 minutes on ice. Cells were washed in 1 ml FACS Buffer, resuspended in FACS Buffer, and then analyzed immediately using a BD FACSCanto II analyzer. For the staining of GMPs in the bone marrow, 1% purified anti-CD16/32(FcγR) was excluded from the ADB and replaced with CD16/32(FcγR)-PE-Cy7. After 15 min on ice, the remaining primary antibodies were added to the cells for 25 min on ice, and the rest of the staining protocol was carried out as described above. F4/80-FITC, CD11c-APC, MHC class II (I-A)-FITC, B220-PE, B220-PE-Cy7, CD69-APC, CD4-PE, CD45.1-FITC, CD3-FITC, CD3-PE, CD8-PerCP-Cy5.5, CD8-PE, GR1-PE, CD115-APC, PDCA1-biotin, PDCA1-APC, CD19-FITC, CD40-PE, c-Kit-FITC, c-Kit-APC, Flt3-PE-Cy5, IL7Rα-PE-Cy7, CD34-FITC, Sca-1-PE-Cy5.5, CD16/32(FcγR)-PE-Cy7, Mac1-PE, Ter119-PE, Sirpα(CD172a)-APC, purified anti-CD16/32, Normal Rat Serum, and Normal Mouse Serum were all purchased from eBioscience. CD86-APC, CD40-PE-Cy7, CD86-PE-Cy7, GR1-APC-Cy7, CD3-PE-Cy7, CD11c-PerCP-Cy5.5, CD11c-PE-Cy7, Mac1-Pacific Blue, CD19-PE-Cy7, CD24-PE, and Sca-1-Pacific Blue were all purchased from BioLegend. CD19-PE and CD3-APC were purchased from Pharmingen. Streptavidin-APC-Cy7 was purchased from BD Biosciences.

### Definitions of identified cell populations

Flow analysis was performed using FlowJo software (TreeStar, Inc). For mice injected with LPS or vehicle: mature spleen cDC were identified as MHCII^hi^CD11c^hi^ cells, spleen B cells were identified as B220+CD3− cells, and spleen T cells were identified as B220-CD3+ cells. Activated B cells and T cells were further defined as CD69+ cells. For steady-state spleen DC and FL-expanded spleen DC: CD8+ cDC were identified as CD3-CD19-PDCA1-GR1-CD11c^hi^Mac1-CD8+ cells, CD8− cDC were identified as CD3-CD19-PDCA1-GR1-CD11c^hi^Mac1+CD8− cells, pDC were identified as Mac1-PDCA1+CD11c^int^ cells, and total cDC were calculated as the sum of CD8+ cDC and CD8− cDC. For bone marrow progenitors: Lineage (Lin) stain contained CD3, CD4, CD8, CD19, Mac1, GR1, B220, and Ter119. KLS were identified as c-kit+Lin-Sca-1+ cells, CLP were identified as Lin-c-kit^lo^sca-1^lo^IL-7R+ cells, CMP were identified as Lin-c-kit+sca-1^lo^CD34+FcγR^lo^ cells, GMP were identified as Lin-c-kit+sca-1^lo^CD34+FcγR^hi^ cells, MDP were identified as Lin-CD115+IL7R-c-kit+Flt3+ cells, and CDP were identified as Lin-CD115+IL7R-c-kit^lo^Flt3+ cells. Additional bone marrow populations: pre-cDC were identified as PDCA1-MHCII-GR1-CD11c+ cells, and pDC were identified as PDCA1+CD11c+ cells. For bone marrow-derived DC cultures: CD8+ cDC equivalent cells were identified as B220-Mac1-MHCII^hi^CD11c^hi^ cells (“Mac1−”) or PDCA1-CD11c+B220-MHCII+CD24+Sirpα− cells; CD8− cDC equivalent cells were identified as B220-Mac1+ MHCII^hi^CD11c^hi^ cells (“Mac1+”) or PDCA1-CD11c+B220-MHCII+CD24^lo^Sirpα+ cells; pre-cDC were identified as PDCA1-CD11c+ MHCII-B220+CD24+ Sirpα− cells; pDC were identified as PDCA1+CD11c^int^ cells. For bone marrow-derived macrophages: Macrophages were identified as F4/80+Mac1+ cells. For mice injected with OT1 CD8+ T cells followed by *L.m.* or control: donor OT1 CD8+ T cells were identified as CD3+CD45.1+CD8+ cells. For mice injected with OT2 CD4+ T cells followed by ova or control: donor OT2 CD4+ T cells were identified as CD3+CD8-CD4+CFSE+ cells.

### In vivo administration lipopolysaccharide

WT and Pin1−/−mice were administered either 300 µl HBSS or 15 mg/kg lipopolysaccharide diluted in 300 µl of HBSS by i.p. injection. Mice were euthanized after 3 or 18 hours.

### In vivo administration of Flt3 Ligand

WT and Pin1−/−mice were administered 1 µg of Flt3 Ligand in 300 µl HBSS by i.p. injection for 9 consecutive days. Mice were euthanized on day 10, and splenocytes were stained and analyzed by flow cytometry.

### Infection with *Listeria monocytogenes* and analysis of CD8+ T cells

Ovalbumin(ova)-specific OT1 CD8+ T cells were obtained from the spleen of an OT1 CD45.1/2 female in a C57BL/6 background using the EasySep CD8+ T cell Enrichment Kit (StemCell Technologies, 19753) according to the manufacturer's protocol. 100 µl of PBS containing 10,000 ova-specific CD45.1/2+ OT1 CD8+ T cells was then injected into the tail vein of each CD45.2+ WT and Pin1−/−mouse. Recombinant *Listeria monocytogenes* (*L.m.*) engineered to secrete chicken ovalbumin was kindly provided by M. Bevan (University of Washington, Seattle, WA) and was prepared as previously described [69]. Twenty-four hours after the injection of OT1 CD8+ T cells, mice were either left uninfected (controls) or infected with 10,000 CFU *L.m.*-ova in 100 µl PBS by tail vein injection. Seven days after *L.m.*-ova infection, mice were euthanized and their spleens removed and analyzed for the presence of CD45.1+ CD8+ T cells by flow cytometry.

### Ova challenge and analysis of CD4+ T cells

Ovalbumin(ova)-specific OT2 CD4+ T cells were obtained from the spleens of OT2 CD45.2 mice in a C57BL/6 background using the CD4+ T cell Isolation Kit II (Miltenyi, 130-095-248). Purified cells were then labeled with CSFE for 5 min at room temperature. 1–2 million cells were injected into WT and Pin1-null mice by tail vein injection. Twenty-four hours after injection of labeled CD4+ T cells, half of the mice received an intraperitoneal injection of 20 ug ova(323–339) resuspended in 200 ul of Sigma Adjuvant System. Forty-eight hours after ova injection, mice were euthanized and their spleens removed and analyzed for the presence of CFSE+CD4+ T cells by flow cytometry.

### Generation of bone marrow-derived dendritic cells and macrophages

FL bone marrow-derived DC (both cDC and pDC) were generated by culturing bone marrow cells at 2×10^6^ cells/ml in DMEM-BM (DMEM containing Pen/Strep, 10% hiFBS, 2 mM L-glutamine, 1 mM Sodium Pyruvate, MEM Non-essential Amino Acids, 10 mM HEPES, and 0.1% β-Mercaptoethanol) supplemented with 50 ng/ml Flt3 Ligand for 9 days. On days 3 and 6, 40% of the media was removed and replaced with an equal volume of DMDM-BM containing 50 ng/ml Flt3 Ligand. On day 9, non-adherent cells were collected for analysis. GM-CSF bone marrow-derived DC were generated by culturing bone marrow cells at 2×10^6^ cells/ml in DMEM-BM supplemented with 5 ng/ml GM-CSF for 5 days. On day 5, non-adherent cells were collected for analysis. Bone marrow-derived macrophages were generated by culturing bone marrow cells at 2×10^6^ cells/ml in DMEM-BM supplemented with 15 ng/ml M-CSF for 6 days. Both adherent and non-adherent cells were collected for analysis. All cultured cells were maintained at 37°C and 5% CO_2_.

### *In vitro s*timulation of bone marrow-derived DC

Bone marrow-derived DC were generated by culturing with Flt3 Ligand for 9 days. On day 9, 100 ng/ml LPS was added to the media for 24 hours. Both adherent and non-adherent cells were collected and analyzed by flow cytometry.

### Immunoblot analysis

Cells were washed in HBSS and then lysed in TritonX Lysis Buffer (150 mM NaCl, 10 mM Tris pH7.4, 10 mM EDTA, 50 mM NaF, and 1% TritonX-100) containing 100 µg/ml Pefabloc (Roche, 11429876001), 10 µg/ml Leupeptin, and 100 nM Okadaic Acid. Lysates were then sonicated and clarified by centrifugation at 10,000 rpm for 5 minutes. Lysates were resolved on 10% or 12% SDS-Page gels and transferred to nitrocellulose. Membranes were incubated in blocking buffer (0.6× PBS containing 0.25% Fish Gelatin, 0.5 mg/ml casein, and 0.02% sodium azide) for ≥1 hour at room temperature, or at 4°C overnight, and then incubated with primary antibodies in blocking buffer containing 0.1% Tween-20 at room temperature for ≥2 hours. Membranes were washed in TBS containing 0.05% Tween-20, and then incubated with secondary antibodies in blocking buffer containing 0.1% Tween-20 and 0.01% SDS for 1 hour at room temperature. After washing, membranes were scanned using the Odyssey Infrared Imager by Li-Cor Biosciences and band intensities were quantified using Odyssey software v3.0. The following commercially available antibodies were used: PU.1 (Cell Signaling, 2266S), IRF8 (Cell Signaling, 5628S), Id2 (Cell Signaling, 3431S), Gfi-1 (Santa Cruz, sc-8558), and β-actin (Sigma, A5441). The Pin1 antibody was generated as previously described [70].

### Cycloheximide analysis

Primary WT or Pin1−/−MEF were grown to 85% confluency in DMEM-MEF (DMEM containing 2 mM L-glutamine, 10% hiFBS, and Pen/Strep). Media was then removed and replaced with either DMEM-MEF alone or DMEM-MEF containing 150 µg/ml cycloheximide reconstituted in water. Cells were incubated for 2–10 hours in the presence of cycloheximide. Both adherent and non-adherent cells were collected, lysed, and analyzed by immunoblot.

### GST pull down assay

The pGEX-2TK vector alone or pGEX-2TK vector containing either WT *Xenopus* Pin1 or a WW domain mutant of *Xenopus* Pin1 (W11A, W34A) were expressed in BL21(DE3) *E. coli* (Stratagene, 200131). Bacteria were lysed in PBS containing 1 mM DTT, 10 mM EDTA, and 100 µg/ml Pefabloc (Roche, 11429876001) and then sonicated. TritonX-100 was added to produce a final concentration of 1% TritonX-100 and lysates were clarified by centrifugation at 12,000 rpm for 20 min at 4°C. GST and GST-Pin1 fusion proteins were then purified by incubating 5 ml of clarified bacterial lysates with 200 µl of a 50/50 slurry of glutathione-agarose beads for 2 hours at 4°C. Beads were washed in Passive Lysis Buffer (PBS containing 1 mM DTT, 10 mM EDTA, and 1% TritonX-100) supplemented with Pefabloc and stored at 4°C. Primary WT and Pin1−/−MEF were grown to 85% confluency in DMEM-MEF (DMEM containing 2 mM L-glutamine, 10% hiFBS, and Pen/Strep) and then lysed in Passive Lysis Buffer (PLB) supplemented with 100 µg/ml Pefabloc, 10 µg/ml Leupeptin, and 100 nM Okadaic Acid. Cell lysates were sonicated and clarified by centrifugation. 1.2 mg of 1 mg/ml lysates were pre-cleared with 20 µg GST beads for 15 minutes at 4°C. 1 mg of pre-cleared lysate was then incubated with 10 µg GST beads, 10 µg GST-Pin1 beads, or 10 µg GST-Pin1WW beads for 2 hours at 4°C. Beads were washed 4 times in 1 ml of PLB, then pelleted, resuspended in SDS-Page Sample Buffer and boiled to elute proteins. Samples were analyzed by immunoblot analysis.

### qRT-PCR analysis

RNA was purified from cells using the RNeasy Mini Kit (Qiagen, 74104) following the manufacturer's protocol. Genomic DNA was removed by on-column digestion with DNaseI using the RNase-free DNase Set (Qiagen, 79254). cDNA was generated from 1–3 µg of total RNA using SuperScript II Reverse Transcriptase (Invitrogen, 18064022). RNA was then removed by digestion with RNaseH (Invitrogen, 18021–071). qRT-PCR analysis was performed using SYBR Green (Invitrogen, 4312704) and the Bio-Rad CFX96 real-time PCR detection system. The following primers were used: PU.1 Forward: 5′-GAGAAAGCCATAGCGATCACTACT GG -3′ and PU.1 Reverse: 5′- ATGTGGCGATAGAGCTGCTGTAG -3′; Cyclophilin A Forward: 5′- GAGCTGTTTGCAGACAAAGT TC -3′ and Cyclophilin A Reverse: 5′-CCCTGGCACATGAATC CTGG -3′.

### Statistical analysis

Data were determined to be statistically significant (*) if p <0.05 by student's t-test. Error bars represent the standard error of the mean.

## Supporting Information

Figure S1 **Spleen B cells and T cells are unaltered in absence of Pin1.** (A) Quantitation of B cells and T cells from WT and Pin1-null mice that were administered vehicle or 15 mg/kg LPS for 18 hours. Cell population numbers were determined by multiplying the frequency of the cell population by the total number of splenocytes obtained from each mouse (n = 3). (B) Quantitation of CD69+ (activated) B cells and T cells from WT and Pin1-null mice that were administered vehicle or 15 mg/kg LPS for 18 hours. Cell population numbers were determined by multiplying the frequency of the cell population by the total number of splenocytes obtained from each mouse (n = 3). (C) Quantitation of cDC from WT and Pin1-null mice that were administered vehicle or 15 mg/kg LPS for 18 hours. Cell population numbers were determined by multiplying the frequency of the cell population by the total number of splenocytes obtained from each mouse (n = 6).
(EPS)

Figure S2 **Frequency of spleen DC subsets in WT and Pin1-null mice.** (A) Frequency of steady-state spleen DC subsets from healthy WT and Pin1-null mice graphed as percentage of total splenocytes (n = 6). (B) Representative FACS plots of WT and Pin1-null steady-state spleen DC subsets. cDC subset plots were previously gated as CD3-19-PDCA1-GR1-CD11c^hi^ cells. pDC plots were previously gated on Mac1- cells. These gates were used to generate data graphed in (A). (C) Splenocytes from WT and Pin1-null mice were counted. Total cell numbers are graphed and the p value is indicated below (n = 8).
(EPS)

Figure S3 **Steady-state dendritic cell development.**
(EPS)

Figure S4 **Frequency of bone marrow populations.** (A) Frequency of bone marrow progenitors in both WT and Pin1-null mice graphed as percentage of total bone marrow cells. KLS, c-kit+Lin-sca1+ stem cells; CLP, common lymphoid progenitor; CMP, common myeloid progenitor; GMP, granulocyte-macrophage progenitor; MDP, macrophage-dendritic cell progenitor; CDP, common dendritic cell progenitor (n = 6). (B) Frequency of pre-cDC and pDC from WT and Pin1-null bone marrow graphed as percentage of total bone marrow cells (n = 6).
(EPS)

Figure S5 **Additional markers identifying cDC equivalents in bone marrow cultures.** Representative FACS plots from WT and Pin1-null bone marrow cells cultured with FL for 9 days. Cells were previously gated as CD11c+PDCA1-B220- and cDC subsets are identified as either CD24+Sirpα− (CD8+ equivalent) cDC or CD24(lo)Sirpα+ (CD8− equivalent) cDC. Frequencies are quantified in the graph at the bottom and shown as percentage of total cells (n = 3).
(EPS)

Figure S6 **Immunoblot analysis of transcription factors involved in DC development.** Transcription factor expression in WT and Pin1-null bone marrow-derived DC and MEF.
(EPS)
